# Genomic non-coding regions reveal hidden patterns of mumps virus circulation in Spain, 2005 to 2015

**DOI:** 10.2807/1560-7917.ES.2018.23.15.17-00349

**Published:** 2018-04-12

**Authors:** Ana M Gavilán, Aurora Fernández-García, Angel Rueda, Ana Castellanos, Josefa Masa-Calles, Noemí López-Perea, María V Torres de Mier, Fernando de Ory, Juan E Echevarría

**Affiliations:** 1 Centro Nacional de Microbiología, Instituto de Salud Carlos III, Majadahonda, Madrid, Spain; 2 Centro Nacional de Epidemiología, Instituto de Salud Carlos III, Madrid, Spain; 3 CIBER de Epidemiología y Salud Pública (CIBERESP), Madrid, Spain

**Keywords:** mumps virus, molecular epidemiology, genotype G, non-coding regions, molecular methods, PCR

## Abstract

Since mumps vaccination was introduced in 1981 in Spain, the incidence of the disease has dropped significantly. However, cyclic epidemic waves and outbreaks still occur, despite high vaccination coverage. The World Health Organization (WHO) recommends genotyping to trace the pattern of mumps virus (MuV) circulation. Genotype H was predominant in Spain, but was replaced in 2005 by genotype G which has subsequently remained dominant. Of the small hydrophobic protein gene sequences, 78% are identical and belong to the MuVi/ Sheffield.GBR.1.05/[G]-variant. **Aim:** Our study aimed to investigate whether the circulation of MuV strains in Spain was continuous after the emergence of genotype G in 2005. **Method:** We obtained 46 samples from Spanish patients infected with MuVi/Sheffield.GBR.1.05/[G] during two epidemic waves and analysed them using new molecular markers based on genomic non-coding regions (NCRs) that discriminate subvariants of this virus strain. **Results:** Phylogenetic analyses of the nucleoprotein–phosphoprotein and matrix protein–fusion protein NCR indicated strain replacement after a drop in incidence in 2009, which had not been detectable by SH sequencing. Clustering of sequences from patients epidemiologically linked in the same outbreak suggests a potential use for these NCRs in outbreak characterisation. **Conclusion:** We suggest to consider their use in conjunction with the SH gene in the future WHO recommendations for MuV epidemiological surveillance.

## Introduction

Mumps is a disease caused by the mumps virus (MuV), which belongs to the genus *Rubulavirus* (family *Paramyxoviridae*). It is an enveloped virus with a 15,384 nt non-segmented, negative-sense RNA genome, with seven transcription units that encode: the nucleoprotein (N), the phosphoprotein (P), the matrix protein (M), the fusion protein (F), the small hydrophobic protein (SH), the haemagglutinin-neuraminidase (HN) and the polymerase (L) [1]. Non-coding regions (NCRs) include the long-terminal repeats (LTRs) at the 3´- and 5´-genomic ends, as well as untranslated regions (UTRs) flanking each coding sequence (CDS) and several intergenic regions (IGs).

MuV is transmitted by direct contact, droplet spread and contaminated fomites. The incubation period is 15–24 days (median: 19 days) [2] and the main clinical manifestations of mumps are fever and swelling of the parotid glands, which occur in 90% of clinical cases. However, the infection is sub-clinical in up to 30% of cases, especially in adults. Orchitis is the most frequent complication in post-pubertal males (20–38%), followed by aseptic meningitis (ca 15%). Other, less frequent complications include oophoritis (0.5–7% of female cases), transient deafness (ca 4%) and encephalitis (ca 0.1%) [3].

Mumps vaccination was introduced into the Spanish childhood immunisation schedule in 1981 as part of the measles, mumps, rubella (MMR) vaccine. The first dose is currently administered at the age of 12 months and the second at 3–4 years [4]. As a result of the sustained increase in vaccination coverage, the incidence dropped from 211 per 100,000 inhabitants in 1982 to 35 per 100,000 inhabitants in 1991 [5]. Although vaccine coverage has exceeded 90% for both doses since 2003, the incidence of the disease has remained between 3 and 31 cases per 100,000 inhabitants since 1997 [5]. The virus still generates outbreaks in Spain and causes cyclic epidemic waves which peak every 4–7 years, most recently in 1996, 2000, 2007 and 2013. This situation is similar in other countries where outbreaks also occur despite high vaccination coverage [6,7]. In fact, many cases occur in vaccinated people [8,9,10]. The cause of this reduced vaccine efficiency remains unclear, but waning immunity [11], incomplete genotype cross-reactivity [12] and antigenic drift [13] have been proposed as explanations.

The World Health Organization (WHO) recommends epidemiological surveillance as a part of the strategy for mumps control [3]. Molecular characterisation of the circulating strains enables identification of circulation patterns and may contribute to the identification of the source of the outbreaks and tracing the transmission chains. Consequently, the WHO has standardised a system for MuV genotyping and a nomenclature for naming strains. Genotyping is based on the sequencing of the SH and HN genes. A total of 12 genotypes have been identified: A, B, C (including the former E), D, F, G, H, I, J, K (including the former M), L and N [3].

Genotype K has been reported in the Basque Country (northern Spain) in historical samples collected during the years 1987 to 1990 [14]. Genotype H was predominant in Spain from 1996 to 2003, but was replaced in 2005 by genotype G which caused the epidemic wave that peaked in 2007 [15] and also subsequent waves [16]. Other genotypes such as A, C, D and J were detected in sporadic cases or in small, limited outbreaks [15], but genotype G has remained predominant from 2005 to the present, with no apparent strain replacement between the last two epidemic waves.

The objective of this work was to investigate whether the circulation of MuV strains in Spain was continuous after the emergence of genotype G in 2005. We evaluated new molecular markers based on the sequencing of NCRs of the MuV genome with the aim of improving the discrimination of genomic variants within genotype G.

## Methods

### Samples and RNA extraction

Among 357 samples from Spanish mumps patients previously characterised as genotype G at the National Center of Microbiology (CNM) according to WHO protocols [16], 78% were MuVi/Sheffield.GBR.1.05/G variant of the MuV genotype G. For this study, we selected 46 of the MuVi/Sheffield.GBR.1.05/G samples that were representative of all years of the study and different geographical origins. Forty-one of them were saliva samples, two were urine samples, two were nasopharyngeal exudates and one was a viral isolate. Thirty samples were collected during the 2005–2009 epidemic period, and 16 were collected during the 2010–2015 wave.

Nucleic acid was extracted with an automatic extractor (QIAsymphony, QIAGEN) using a commercial kit (QIAsymphony Virus/Bacteria Midi Kit (96); Qiagen). 400 µL of sample was extracted to obtain 40 µL of final eluate.

### Selection of genomic regions, primer design and amplification conditions

Previous studies of MuV genome variability have shown the NCR located between the N and P CDS (N-P), the P and M CDS (P-M) and the M and F CDS (M-F) to be the most variable, together with the SH gene recommended by the WHO for genotyping [17]. Fifty-five complete genomes of eight different MuV genotypes (A, B, C, G, H, I, K, N) were taken from the GenBank database and aligned with MAFFT v.7 [18]. Consensus primers flanking the NCR were designed from the alignment (Table) to amplify fragments containing these regions by nested RT-PCR.

**Table:** Primers used for RT-PCR

Primer name^a^	Sequence (5’–3’)	Location (nt 5'–3´)^b^	Annealing temperature (°C)	Fragment length^c^
NP_F1	CATGCCAyTATATCCTCAAGTCAG	1,591–1,614	57	923
NP_R1b	GCATATrGGGTTGCACCACT	2,557–2,538		
NP_F2	GATTTATTACGATACAACGArAAyGG	1,673–1,698	55	774
NP_R2	TCTATGCCCTCCTCTTGGA	2,491–2,473		
PM_F1	GCTACAATTGAAGGAATGATGGC	2,766–2,788	60	859
PM_R1	TTGGGGATGCGGTTGATCT	3,667–3,649		
PM_F2	GTCCCAGTTGATGAGCTTAGAAG	2,826–2,848	55	759
PM_R2	ACTyGCTGTCTTCCGAACC	3,626–3,608		
MF_F1	TCATCACCATCGTTrGCGAA	4,185–4,204	55	504
MF_R1	GTTATCAGTGGGTTGGATATTyGG	4,731–4,708		
MF_F2	TGGTCATCTGGGTGTGAAATC	4,212–4,232	55	428
MF_R2	GAGCTTGAACTTTGTGAGTAATAGC	4,685–4,661		

nt: nucleotide.

^a^ NP, PM, MF: name of fragment containing the corresponding NCR; F: forward; R: reverse; 1: primary reaction; 2: nested reaction.

^b^ MuVi/Gloucester.GBR/32.96/ (AF280799) was used as reference sequence.

^c^ Length excluding primer sequences is presented here.

The OneStep RT-PCR kit (QIAGEN, Hilden, Germany) was used for reverse transcription and primary amplification, while nested PCR was performed using the BioTAQ DNA polymerase (Bioline, London, United Kingdom). The reaction mixture contained 0.6 µM (N-P and P-M) or 1 µM (M-F) of each primer (forward and reverse) and 400 µM (N-P and P-M) or 600 µM (M-F) of dNTPs and 2.5 mM of MgCl_2_. The thermal profile was 50 °C for 30 min, 95 °C for 15 min, then 40 cycles of 94 °C for 30 s, annealing temperature (Table) for 60 s, 72 °C for 90 s (N-P and P-M) or 60 s (M-F), followed by a final extension step at 72 °C for 10 min. The nested reaction mixture contained 0.4 µM of each primer, 200 µM of dNTPs and 2 mM of MgCl_2_. The thermal profile was 94 °C for 2 min, then 30 cycles of 94 °C for 15 s, 55 °C for 30 s and 72 °C for 80 s (N-P and P-M) or 60 s (M-F), followed by a final extension step at 72 °C for 7 min. 

### Sequencing and phylogenetic analysis

PCR products were purified by enzymatic reaction Illustra ExoProStar 1-Step (GE Health Care Life Science, Freiburg, Germany) and sequenced with the ABI Big Dye Terminator Cycle Sequencing Kit (Applied Biosystems, Branchburg, NJ, United States) using the corresponding forward and reverse primers. Sequences were edited using BioEdit v.7.2.5 [19] and aligned with MAFFT v.7 [18]. Haplotypes (a set of identical sequences) were identified using DNAsp v5 software [20]. Phylogenetic analysis was performed by maximum likelihood method with the RaxML programme [21] through the BlackBox website (http://phylobench.vital-it.ch/raxml-bb). To confirm the results, the PhyML 3.0 programme [22] was used through the ATGC portal (http://atgc.lirmm.fr/phyml/). The best evolutionary model was previously selected using jModelTest v.2.1.10 [23] according to the Akaike information criterion. Sequence fragments of the various genes were concatenated using Seaview v.4 [24] and in this case PartitionFinder v. 1.1.1 [25] was used to select the evolutionary model. Phylogenetic trees were edited using MEGA v.6.06 software [26].

### GenBank accession numbers

The sequences obtained in this study have been deposited in GenBank under accession numbers KY607678-KY607722 for N-P NCR, KY607632-KY607677 for P-M NCR, and KY607586 - KY607631 for M-F NCR.

## Results

### Analysis of sequence variants

**Figure 1:**
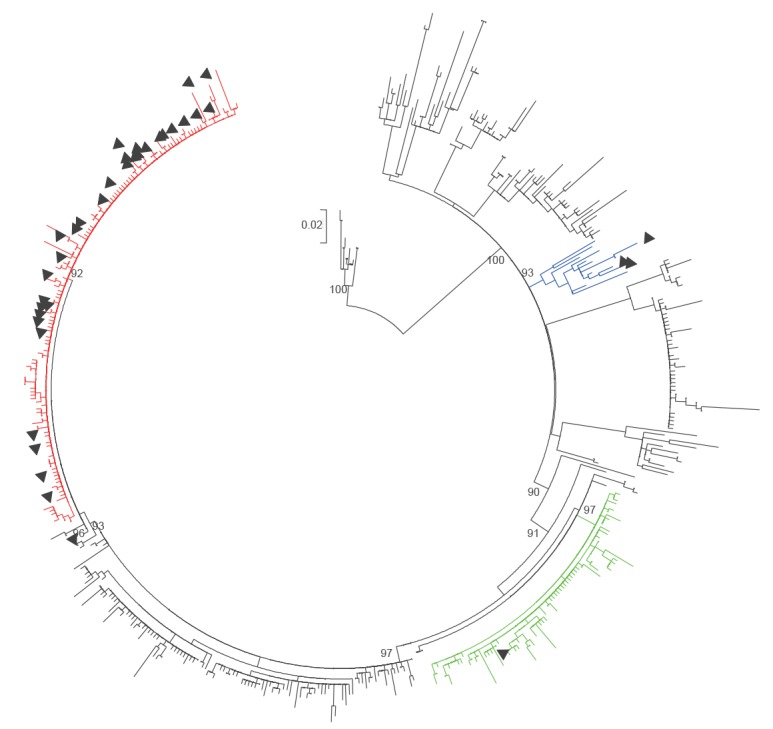
Phylogenetic tree of mumps virus SH haplotypes of genotype G

A total of 1,292 samples positive for MuV RNA were identified by RT-PCR at the CNM between 2005 and 2015 [16]. The genotype was studied in 591 of them and 543 (91.9%) proved to belong to genotype G. The full SH gene of 357 of them was available for variant characterisation, from which 30 distinct haplotypes were identified (Figure 1).

The tree was created with the maximum likelihood method in PhyML, using one sequence of each haplotype of all the sequences from GenBank (until June 2016) and Spanish sequences (January 2005 to June 2016). The GTR+G model of evolution was used and the αLRT SH-like value was analysed. Spanish sequences are indicated with a black triangle. Only support values of αLRT SH-like > 90 are shown. Clades including Spanish sequences are shown in colour. The clade comprising the MuVi/Sheffield.GBR /1.05 [G] variant and its related haplotypes is shown in red.

278 samples (78%) belonged to the same haplotype, displaying an identical SH sequence that was also identical to 346 of the 1,535 sequences from other countries that were available in GenBank. The WHO systematic name of the oldest sample was chosen to name the variant (MuVi/ Sheffield.GBR.1.05/[G]) [3].

### Ability of non-coding regions to discriminate the SH subvariants of MuVi/ Sheffield.GBR.1.05/[G]

In 45 of the 46 selected samples, we successfully amplified and sequenced all three NCR fragments. The N-P containing fragment could not be amplified in one sample. Nested amplification was not required for the amplification of the M-F region in 16 samples that showed a clear band after the first reaction. The length of the fragments used for phylogenetic analysis was 768 nt (N-P), 758 nt (M-P) and 428 nt (M-F).

**Figure 2:**
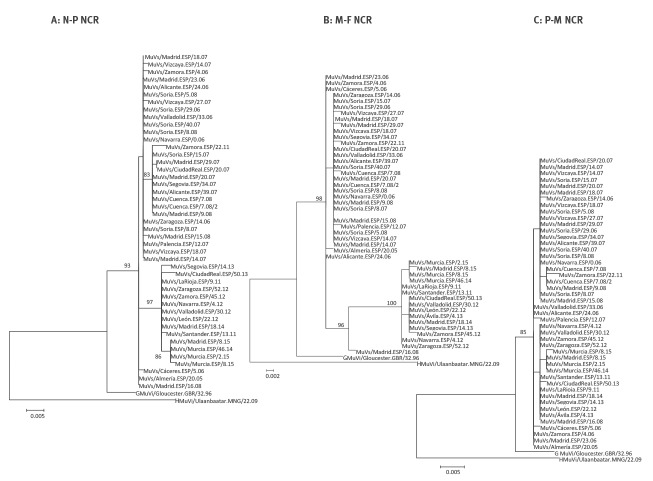
Phylogenetic trees of mumps virus sequences containing non-coding regions, Spain, 2005-2015 (n = 46)

HKY: Hasegawa, Kishino, Yano; MuV: mumps virus.

The sequence containing N-P showed 27 variable positions (one per 28.4 nt, 96.4% conserved positions), while the sequence containing P-M had 11 (one per 68.8 nt, 98.5% conserved positions). Finally, the sequence containing M-F proved to be the most variable with 23 variable positions (one per 18.6 nt, 94.6% conserved positions). According to this, M-F and N-P regions both enabled discrimination of different groups in the phylogenetic tree, while well sustained clades were not observed for the P-M region (Figure 2). Consequently, we decided to proceed with the concatenation of the M-F and N-P sequences, but not the P-M sequences.

Analysis was carried out using the maximum likelihood method in PhyML and HKY as the evolutionary model. Statistical analysis involved the use of a bootstrapping approach. The MuV genotype G reference sequence was included (MuVi / Gloucester.GBR / 32.96 [G]), and MuVi / Ulaanbaatar.MNG / 22.09 [H] was used as the outgroup.

**Figure 3:**
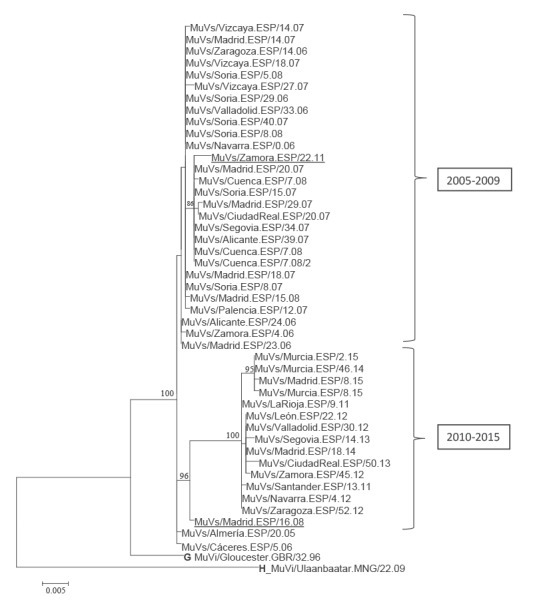
Phylogenetic tree of N-P and M-F concatenated sequences, Spain, 2005-2015 (n = 45)

HKY: Hasegawa, Kishino, Yano; MuV: mumps virus.

As a consequence of the concatenation of the N-P and M-F NCR regions, it was possible to distinguish 24 haplotypes in selected 45 samples belonging to the MuVi/ Sheffield.GBR.1.05/[G] variant (Figure 3).

The tree was derived with the maximum likelihood method in PhyML, using the HKY as the evolutionary model, and a bootstrapping approach for the statistical analysis. The MuV genotype G reference sequence was included (MuVi / Gloucester.GBR / 32.96 [G]) and MuVi / Ulaanbaatar.MNG / 22.09 [H] was used as the outgroup. Bootstrap values > 70 are shown. The two sequences that clustered with sequences from a different time period are underlined.

### Variant circulation

The NCR variants detected in samples from 2005 to 2009 and those circulating from 2010 to 2015 formed different phylogenetic groups (Figure 3). The results were similar irrespective of whether they were based on the N-P or the M-F region (Figure 2, panels A and B). Two of the earliest strains (MuVs/Almería.ESP/20.05, MuVs/Cáceres.ESP/5.06) were the closest to the root of the tree. A well-supported subgroup of four sequences was observed within the 2010 to 2015 clade, obtained from patients involved in the same outbreak in the city of Murcia. Two exceptions to this temporal classification were observed: the MuVs/Zamora.ESP/23.11 strain isolated in 2011 grouped with those from 2005 to 2009. The opposite was the case for MuVs/Madrid.ESP/16.08 isolated in 2008, although this strain was located in a basal position in relation to the 2010 to 2015 clade.

## Discussion

The main limitation of this study is that it was restricted to a single genotype (G). It would have been particularly interesting to reproduce the results on the genotype dominant before 2005 (genotype H), but we could not obtain enough material from sample collections.

Genotype G was the most frequently detected genotype in Spain during the period of the study, as well as in other western countries and Japan [3,17,27]. A particular variant of this genotype (MuVi/ Sheffield.GBR.1.05/[G]) seemed to be dominant, not only in Spain but also in other countries, according to GenBank records. Such dominance of a single variant suggests continuous circulation of MuV [G], despite the sharp drop in incidence registered in Spain during the study period between the two epidemic waves [5]. In contrast, interruption of transmission of genotype H after a previous drop in incidence in 2004 was followed by the emergence of genotype G [5,15].

The WHO recommend genotyping MuV strains on the basis of the SH sequences in order to identify the patterns of circulation [3]. However, the SH region does not always provide enough resolution, and other genes such as H and F have been proposed as new markers to complement SH [28]. A recent study showed the NCR regions N-P, P-M and M-F, in addition to the SH gene, to be the most variable regions of the MuV genome [17]. Moreover, in the case of genotype G, the M-F NCR region seems to be more variable than the SH gene [17]. Our results are consistent with this conclusion. We found the M-F region to be the best molecular marker to complement the SH region in molecular epidemiology studies of the dominant genotype G. A short fragment of 428 nt that includes this region proved to be easily amplified and sequenced by the proposed protocol, which makes it very suitable for the routine tasks carried out by most national reference laboratories into epidemiological surveillance and outbreak management of mumps in real time. However, according to a previous study [17], circumstances could be different for other genotypes, so this needs to be investigated further. In this context, the sequences containing NCR region N-P proved to be a useful complement if additional discriminatory power is required.

The analysis of the sequences containing NCR variable regions M-F and N-P clearly revealed a strain replacement after the drop in incidence in 2009, which remained hidden after SH sequencing. The group of strains that subsequently emerged could be minor components of the viral populations circulating previously that would have been selected through a bottleneck mechanism because of decreased incidence. The evolutionary relationship between the two clades suggested by the phylogenetic tree topology, and the basal position of the strain MuVs/Madrid.ESP/16.08 in the 2010 to 2015 clade seem to support this hypothesis. Alternatively, they could have been imported from another geographical location after extinction of the previously circulating strains. Importation of other genotypes that cause sporadic cases and small outbreaks has been observed in the context of the circulation of genotype G [15]. Moreover, a genotype G strain (MuVs/Zamora.ESP/23.11) genetically related to those circulating from 2005 to 2009 was detected in 2011, suggesting either that it was imported from other parts of the world where these ancestral strains remained active after disappearing from Spain or that it continued circulating at low level. Additional data from the NCR regions of strains detected in other countries are needed to verify the importation hypothesis. Finally, the subgrouping of some sequences obtained from patients epidemiologically linked in the same outbreak suggests a potential use for these NCR regions in outbreak characterisation and discrimination of transmission chains. This merits further exploration.

We suggest that it would be worthwhile considering the use of these NCR regions, particularly M-F in genotype G, along with the SH gene, in the WHO’s future recommendations for the epidemiological surveillance of MuV.
